# Supplementary material for: When access ≠ acceptance: How clinical specialty demands shape mutual recognition on medical examination/test result reuse in Chinese hospitals: A study on a pilot province of China’s medical digital reform
Source: PLoS One. 2025 Oct 15;20(10):e0318899. doi: 10.1371/journal.pone.0318899 (PMC12527183; doi:10.1371/journal.pone.0318899)
Supplement: Attachment — Submitted filename: 2593 Report.docx [file pone.0318899.s002.docx]

**Review of “Digital healthcare reform and reduction of duplicate examinations and tests: a study of physicians' behavior on mutual recognition in response to clinical specialized demands and information intervention form a national pilot province in China”**

**General Notes:**

- The introduction lacks a clear articulation of the research gap. It does not sufficiently justify the necessity of this specific study or differentiate it from existing research in the field. The framing of the problem is vague, and the significance of studying mutual recognition indicators in this context is not convincingly established.
- The methodology section presents several limitations that undermine the credibility of the study. While it is stated that the study involves eight hospitals, the selection criteria for these hospitals are not well justified. There is no indication of whether these hospitals are representative of the larger healthcare system in China. Additionally, the study relies on a set of recognition indicators, but these indicators are not adequately validated. The definitions provided for these metrics lack clarity, and there is no discussion of their reliability or potential biases. The statistical methods used for analysis are also not sufficiently detailed.
- The results section is overly descriptive and does not provide deep analytical insights. The study reports various recognition indicators across hospitals and clinical specialties, but it does not establish meaningful connections between these variables. The discussion fails to interpret the significance of the findings in a broader context. There is little attempt to compare the results with previous studies, making it unclear how this study contributes to the existing literature. The authors mention differences in physician behavior across hospitals and specialties but do not provide a strong theoretical framework to explain these variations.
- A significant limitation of the study is its lack of consideration for potential confounding factors. The recognition rates and behaviors observed may be influenced by numerous external variables such as hospital policies, physician workload, patient demographics, and technological infrastructure. However, the study does not adequately control for these factors, making the conclusions unreliable. Furthermore, the authors make broad claims about the impact of mutual recognition policies on hospital management without providing sufficient empirical evidence.
- The manuscript’s writing and organization also present issues. There are numerous instances of awkward phrasing, grammatical errors, and unclear sentence structures that hinder readability. Some sections are repetitive, particularly in the explanation of recognition indicators. The discussion does not flow logically, and key points are often buried within dense paragraphs. Additionally, the figures and tables, while potentially useful, are not well integrated into the narrative, making it difficult to understand their relevance to the main argument.
- The ethical considerations in the study are inadequately addressed. The authors mention that the data were anonymized, but they do not provide details about institutional ethical approvals or patient consent procedures. Given that the study involves physician behavior in a clinical setting, ethical approval should be explicitly stated.
- Lastly, the study should offer substantial practical implications. The conclusion is broad and does not provide clear recommendations for policymakers, hospital administrators, or clinicians. While the authors emphasize the importance of mutual recognition in digital healthcare, they do not suggest concrete ways to improve implementation based on their findings. This weakens the study’s potential impact and relevance to real-world applications.

**Introduction**

- The introduction lacks a strong and clear research gap, making it difficult to understand the necessity of the study. While it introduces the concept of mutual recognition (MR) in medical testing, it does not sufficiently differentiate this research from previous studies. There is no explicit discussion of how this study advances the existing body of knowledge or addresses a specific problem that remains unresolved. The background information provided is broad, and the study’s significance is not convincingly justified.
- There are multiple instances where claims are made without proper citations. For example, the statement that "The Diagnosis-Related Groups (DRG) payment method controls medical expenses by setting predetermined cost standards, encouraging hospitals to use resources efficiently" lacks a supporting reference. Given the significance of this claim, a proper citation is necessary to substantiate the argument. Similarly, the claim that "The MR had significantly reduced the unnecessary repetitive examinations and tests when patients seek treatment across different hospitals in the past three years" requires empirical support. Without proper references, these statements appear as unsupported assumptions rather than evidence-based facts.
- Some references are either mismatched or incorrect. The introduction states that "Zhejiang Province has taken the lead in establishing the 'Zhejiang Medical Mutual Recognition' platform (MR platform) in 2021" but does not provide a valid reference for this claim. The citation provided later in the document does not specifically support this assertion, leading to a potential mismatch between the reference and the information presented. Furthermore, the claim that "On the government website of Zhejiang Province in China, as of 2022, the MR platform had cumulatively recognized various items 16.81 million, directly saving medical expenses of 712 million RMB" is presented without a proper citation, making it difficult to verify the accuracy of these numbers.
- Several sentences contain grammar and language mistakes that affect clarity and readability. For example, the phrase "The MR reduces unnecessary duplicate examinations and tests, thereby lowering costs" should be revised to "MR reduces unnecessary duplicate examinations and tests, thereby lowering costs." The definite article “The” is unnecessary in this context. Another example is "Since the MR platform's launch three years ago, by the end of 2024, it has been deeply integrated into clinical practice." The phrase "by the end of 2024" suggests a future event, yet the sentence is structured in the past tense, making it inconsistent. Additionally, the phrase "has significantly reduced diagnostic cycles, markedly improving the efficiency and quality of medical services, and effectively reducing the burden on patients" is redundant. The repetition of "reducing" should be streamlined for clarity.
- Certain statements in the introduction lack neutrality and objectivity, making them sound promotional rather than scientific. For instance, the description of the MR platform as "dedicated to breaking down barriers in medical data and achieving seamless MR and efficient sharing of examination and test results" is overly enthusiastic and lacks empirical validation. A more neutral and evidence-based phrasing would enhance the credibility of the study.
- The introduction also fails to properly define key terms and concepts. For instance, while mutual recognition and its relevance to digital healthcare reform are mentioned, there is no clear operational definition provided. The study assumes that the reader is familiar with the intricacies of MR policies, but a more structured explanation would be necessary for clarity. Similarly, there is no clear distinction made between mutual recognition at different levels of the healthcare system, which could lead to confusion.
- There is inconsistency in terminology and capitalization. The phrase "traditional chinese medicine" is used multiple times, but "Chinese" should be capitalized. Additionally, terms such as "Medical Mutual Recognition" are sometimes written with inconsistent capitalization, making the text appear unpolished.
- Overall, the introduction is poorly structured and lacks logical flow. Ideas are introduced without a clear connection, and transitions between paragraphs are weak. Some sections are overly detailed with numerical data that could be more effectively presented in the results section rather than in the introduction.

**Methods**

- The Methods section contains multiple issues related to clarity, validity, and proper referencing, which undermine the reliability of the study. One of the major flaws is the lack of justification for the hospital selection process. The manuscript states, “This study was a multicenter analysis, including various types of hospitals across China, all of which were large tertiary hospitals (grade A hospitals), and were concentrated in the provincial capital city of Hangzhou.” However, there is no explanation of why only tertiary hospitals were included, or whether these hospitals are representative of the entire healthcare system in China. The absence of selection criteria introduces a potential selection bias, making it unclear whether the findings can be generalized beyond these specific institutions.
- The study mentions that data were collected from “a total of eight hospitals” without specifying whether these hospitals were randomly selected or chosen based on predefined criteria. Moreover, it is stated that these hospitals were selected based on “the scale of their operations, with each exceeding an annual medical revenue of 1.6 billion RMB (approximately 225 million USD) in 2023.” However, no reference is provided to support this claim or validate the accuracy of these financial figures.
- The description of data collection methods is vague and lacks necessary details. The manuscript states that “The physician behaviors of eight hospital were recorded by the MR platform, which was accessible to hospitals.” However, it does not clarify how these behaviors were recorded, whether physicians were aware of the data collection, or whether the data collection process had any impact on their decision-making. Furthermore, it is unclear if any bias was introduced due to the presence of the MR platform, which could influence physician behavior differently across hospitals.
- The methodology for defining recognition indicators lacks proper validation. The study introduces a set of indicators such as "Total Recognition Proportion (TRP)," "Total Recognition Rate (TRR)," and "Precision Delivery Rate (PDR)" but does not provide any prior literature or references supporting their use. These indicators appear to be novel but are not validated against existing models or frameworks. Additionally, the manuscript states, “The definitions and connotations of each recognition indicator were detailed as flowers,” which appears to be a typographical or grammatical error that disrupts readability. This phrase is unclear and should be properly revised to indicate where and how the definitions are provided.
- There are multiple issues with the explanation of the mutual recognition workflow. The manuscript states, “If the physician consistently overlooks the delivered reports, the item order can also be executed by the local hospital.” However, the term “consistently overlooks” is vague and lacks a quantitative threshold—how many instances of overlooking a report are considered "consistent"? Furthermore, there is no discussion on how physician decisions were categorized, whether subjective judgment played a role, or whether any measures were taken to account for physician variability in interpreting mutual recognition data.
- The study describes an information intervention implemented in three hospitals but fails to explain how the intervention was standardized and whether it was controlled for potential confounding factors. The manuscript states, “Hospitals A, C, and F carried out the information intervention on path of overlooked access while other hospitals had not set up.” However, there is no explanation of why these specific hospitals were selected for intervention, or whether their baseline characteristics differed from the control hospitals.
- Ethical considerations are inadequately discussed. The manuscript states, “All the data were subjected to anonymization.” However, there is no mention of whether the study was approved by an institutional review board (IRB) or ethics committee. Additionally, there is no information about whether informed consent was obtained from the physicians whose behaviors were analyzed. Given that the study involves physician decision-making, ethical approval and participant consent should have been explicitly documented.
- There are grammatical issues throughout the Methods section, which affect readability. For instance, “Hospital management strategies on recognition had an affect on physicians’ behaviors” should be corrected to “Hospital management strategies on recognition had an effect on physicians’ behaviors.” Similarly, the phrase “The comparisons at the clinical specialty level began by categorizing data into four major groups” is unclear—it should specify what the four major groups are before proceeding with the analysis. Additionally, phrases like “To further delineated the behavioral differences among clinical specialties” should be corrected to “To further delineate the behavioral differences among clinical specialties” for grammatical accuracy.

**Results**

- The authors should modify the Results section, as it presents multiple issues that undermine its validity, clarity, and reliability. One of the major concerns is the excessive use of descriptive statistics without any deep analytical insights. The section reports various recognition indicators across different hospitals and clinical specialties but fails to provide a critical interpretation of these numbers. For example, the manuscript states, “During the study period, the 8 large hospitals received a total of 26,759,077 delivered items through the MR platform, with 3,639,368 precision-delivered items sent to meet physicians' orders.” While this provides raw data, it does not explain the significance of these numbers or how they compare to prior studies or benchmarks. The authors do not discuss whether these figures indicate success or inefficiencies in the MR platform.
- A major issue is the lack of statistical validation. While several metrics such as Total Recognition Proportion (TRP) and Cross-Hospital Recognition Rate (CHRR) are reported, there is no indication of whether these differences are statistically significant. For example, the manuscript states, “The traditional Chinese medicine demonstrated a low cross-hospital precision delivery rate but a high rate of recognizing reports from other hospitals, contrasting with pediatrics.” However, the authors do not provide p-values, confidence intervals, or effect sizes to support this claim. Without proper statistical tests, it is unclear whether the reported differences are meaningful or simply due to random variation.
- Some of the reported figures appear to lack proper references or supporting explanations. For instance, the manuscript states, “The total recognition proportion for the traditional Chinese medicine group and the pediatrics group were 51.18% and 9.95%, respectively.” However, there is no comparison with other studies or previously established benchmarks. Additionally, it is unclear whether these numbers are high or low in a broader context. The study does not explain whether such variations are expected in clinical practice or if they signal a problem with the MR platform.
- There are inconsistencies and contradictions within the results. The manuscript claims that “the differences were not significant among the same clinical specialties in different hospitals,” but then later states that “some recognition indicators were also noticeably affected by the mutual recognition management strategies of the affiliated hospitals.” This contradiction suggests a lack of clarity in the findings. If hospital management strategies do affect recognition indicators, then it is misleading to claim that differences across hospitals are insignificant. The authors need to clarify their findings and ensure consistency in their interpretation.
- Some sections contain incorrect or misleading phrasing. The manuscript states, “Information intervention had increased the workload for physicians in accessing duplicate reports, mainly stemming from the repetitive process of accessing reports from their own hospital both locally and on the platform, without significant affect on the main recognition indicators across hospitals.” The phrase “without significant affect” contains a grammatical error—“affect” should be replaced with “effect” in this context. Additionally, the claim that the intervention increased physician workload lacks quantitative evidence. The authors should have measured physician workload (e.g., through time tracking or surveys) and reported this data instead of making a vague assumption.
- Some key results are not well integrated into the discussion. For instance, the manuscript mentions, “The highest rates in both TRP (75.77%) and CHRP (48.96%) were shown in Hospital D, indicating strong policy adherence and intra-hospital collaboration.” However, there is no exploration of why Hospital D performed better than others. Did Hospital D have different training programs, superior IT infrastructure, or other distinguishing factors?
- The presentation of figures and tables is problematic. The manuscript includes statements such as, “Table 1 shows the recognition indicators across clinical specialty groups in the MR platform,” but does not provide a summary of the key takeaways from the table. Simply listing numbers without interpretation does not provide useful insights. Additionally, some table labels and column names are vague. For instance, “PDR” (Precision Delivery Rate) is introduced in a table, but its role in influencing physician behavior is not well explained. The authors should include a brief interpretation for each table to make it more accessible.
- There is a lack of proper citations in the Results section. While the study introduces several findings about physician behavior and mutual recognition, it does not reference any prior research to support these findings. For example, the manuscript claims, “The positive correlation observed between the OAR and TRP implies that physicians with a higher rate of overlooking access reports also exhibit a higher TRP.” However, without a reference to previous studies or an explanation of why this relationship exists, the claim remains speculative. The authors should compare their findings to existing literature and discuss whether their results align with or contradict prior studies.
- The language used in the Results section is often unclear and redundant. For instance, the phrase “The correlation between CHRP and TRP, presenting a positive correlation (r=0.776, p<0.05)” is awkwardly phrased. A clearer way to express this would be, “A significant positive correlation was found between CHRP and TRP (r=0.776, p<0.05).” Another example is the phrase “These analyses collectively aimed to provide a comprehensive understanding of how recognition practices, cost implications, and overlooked access interacted within and across hospitals.” The phrase “interacted within and across hospitals” is unnecessarily repetitive and could be simplified.
- Finally, the section fails to provide practical implications for the findings. While a variety of recognition indicators are reported, there is little discussion of how these results could inform hospital policies or physician training. The authors do not offer recommendations for improving recognition rates or reducing overlooked reports. A more robust discussion of how hospitals can use these findings to enhance efficiency would improve the impact of the study.

**Discussion**

- The Discussion section of this manuscript presents several fundamental weaknesses that undermine the credibility and impact of the study. One of the most significant issues is the lack of connection between the results and the broader literature. The discussion does not effectively compare the study’s findings with previous research, making it unclear how the results fit within the existing body of knowledge. For example, the manuscript states, “The implementation of DRG reform in China's healthcare system has significantly influenced the necessity and affect of MR.” However, this claim is made without proper citation or discussion of specific studies that support this argument. Without references, this statement appears as an unsupported assertion rather than a well-founded conclusion.
- There is misuse of references and mismatched citations throughout the discussion. For instance, the manuscript states, “This integrated approach is crucial for the future of healthcare management and policy-making in China, as it tackles the challenges of escalating healthcare costs and the demand for high-quality, cost-effective medical services.” While this is a strong claim, no reference is provided to support it. The discussion lacks citations when making claims about the importance of mutual recognition (MR) in healthcare policy, making the argument appear speculative rather than evidence-based.
- The language and grammar errors in this section further reduce the clarity of the arguments. For example, the manuscript states, “The MR had significantly reduced the unnecessary repetitive examinations and tests when patients seek treatment across different hospitals in the past three years.” This sentence contains a verb tense inconsistency, as “had significantly reduced” is in past perfect tense, while “patients seek treatment” is in present tense. The sentence should be corrected to: “MR has significantly reduced unnecessary repetitive examinations and tests when patients seek treatment across different hospitals over the past three years.”
- There are contradictions in the interpretation of the results. The manuscript claims, “Hospital management strategies, such as information interventions, need to strike a balance between mutual recognition work and the workload of physicians accessing duplicate reports.” However, earlier in the Results section, the study indicates that information interventions significantly reduced overlooked access rates but did not affect overall recognition rates. This contradiction raises concerns about the internal consistency of the study’s conclusions. The authors should provide a clear and logical synthesis of the findings rather than presenting conflicting statements.
- The discussion contains vague and unsupported generalizations. For example, the manuscript states, “By conducting an in-depth analysis of recognition indicators from various large hospitals, this study provides reliable evidence and strong guidance for precisely identifying differences in the performance of MR across different clinical specialties.” This is a broad statement that lacks specific supporting data. While the study reports recognition indicators across hospitals, there is no justification for calling the findings “reliable evidence” or “strong guidance” without demonstrating how the conclusions can be generalized beyond the study sample.
- There is redundant and unclear phrasing throughout the discussion. The phrase, “The recognition indicators constructed in this study offers significant analytical and evaluative value for the recognition behaviors of physicians on the MR platform,” is grammatically incorrect because “offers” should be “offer” to match the plural subject “indicators.” Moreover, the phrase is redundant, as “analytical and evaluative value” essentially mean the same thing. A clearer way to state this would be: “The recognition indicators developed in this study provide valuable insights into physician behavior on the MR platform.”
- The discussion fails to provide actionable recommendations based on the findings. While the manuscript presents hospital recognition strategies and mutual recognition trends, it does not offer concrete steps for hospital administrators, policymakers, or clinicians to improve mutual recognition processes. The authors should provide specific strategies for optimizing hospital workflows, improving information intervention mechanisms, or refining recognition indicators, but these aspects are either missing or vaguely mentioned.
- The limitations of the study are inadequately discussed. The manuscript acknowledges some limitations but fails to address critical methodological concerns. For example, the study does not discuss potential confounding factors such as physician experience levels, hospital policies, or technological differences in data-sharing platforms. The discussion states, “There were other management methods that could also affect the recognition indicators of clinical specialties, such as improving the quality of various exams and tests, conducting promotional training, and performance assessments.” However, it does not analyze whether these factors influenced the study’s results. A stronger discussion of these limitations would improve the transparency and credibility of the study.
- The future research section is superficial and lacks specific directions. The manuscript states, “Future research can be carried out in the following areas: First, extend the data collection period and increase the participating hospitals to more accurately grasp the long-term trends and patterns of change.” While this is a reasonable suggestion, it lacks depth. The authors should propose specific research methodologies, such as longitudinal studies or randomized trials, to strengthen future investigations. Additionally, the suggestion to "increase the participating hospitals" does not address whether additional hospitals would introduce new variables or require more advanced statistical methods to control for differences.
